# The Effectiveness of Nurse-Led Multidimensional Digital Cardiac Rehabilitation in Patients With Unstable Angina Undergoing Percutaneous Coronary Intervention: Emulated Target Trial

**DOI:** 10.2196/75325

**Published:** 2025-08-27

**Authors:** Tong Zhou, Yijun Wang, Jun Wang, Jinjun Liu, Nana Zhang, Xiaoling Zhang, Shengnan Yao, Mingming Tang, Guixia Xu, Yongxia Chen

**Affiliations:** 1 Department of Cardiology, The First Affiliated Hospital of Bengbu Medical University, Bengbu, China; 2 West China Hospital, Sichuan University, Chengdu, China; 3 Joint Research Center for Regional Diseases of IHM, The First Affiliated Hospital of Bengbu Medical University, Bengbu, Anhui, China; 4 Department of Nursing, The First Affiliated Hospital of Bengbu Medical University, No. 287, Changhuai Road, Longzihu District, Bengbu, 233000, China, 86 05523086107

**Keywords:** unstable angina, percutaneous coronary intervention, digital health technologies, cardiac rehabilitation, Baduanjin, mHealth, multicomponent intervention, multidimensional intervention

## Abstract

**Background:**

Cardiac rehabilitation (CR) interventions for patients with coronary heart disease are increasingly adopted. However, research on the integration of digital health technologies into CR for patients with unstable angina (UA) undergoing percutaneous coronary intervention (PCI) remains limited.

**Objective:**

This study assessed the effectiveness of a multidimensional digital CR program for patients with UA undergoing PCI.

**Methods:**

This prospective study enrolled 164 patients with UA who underwent PCI between April and June 2022. Patients were assigned to either the usual care group (April-May 2022) or the multidimensional digital CR intervention group (May-June 2022). The intervention group received rehabilitation through a nurse-led, multidisciplinary team, using a customized digital CR program. This program encompassed 7 key rehabilitation components: exercise, medication management, nutritional guidance, psychological support, sleep management, health education, and smoking cessation assistance. The usual care group received standardized treatment and routine nursing care. To minimize selection bias, propensity score matching was applied between the 2 groups. The primary outcomes included changes in the 6-minute walk test (6MWT), 12-item Short Form Health Survey (SF-12) scores, and frailty phenotype scores at 3 months. Secondary outcomes assessed differences in gait speed, 30-second chair stand test (30-s CST), grip strength, waist circumference (WC), BMI, and lipid profiles at 3 months.

**Results:**

A total of 136 patients were included in the final analysis. At 3 months, the intervention group demonstrated significant improvements in frailty status compared to the control group. The proportion of prefrail patients decreased from 100% (68 patients) to 75% (51 patients), while nonfrail patients increased from 0% to 25% (17 patients; *P*<.001). Regarding physical fitness, the intervention group exhibited improvements in 6MWT: from 347.06 (SD 32.43) to 375.22 (SD 29.71) m (*P*<.001); gait speed: from 0.87 (SD 0.17) to 1.05 (SD 0.14) m/s (*P*<.001); and 30-s CST: from 10.0 (SD 1.89) to 12.71 (SD 1.97; *P*<.001). Grip strength, BMI, and WC improved significantly in the intervention group. Grip strength increased from 16.64 (SD 6.57) to 20.74 (SD 5.37; *P*<.001). BMI decreased from 25.74 (SD 3.05) to 23.88 (SD 2.14; *P*<.001), and WC decreased from 94.98 (SD 7.87) to 89.91 (SD 7.50) cm (*P*<.001). The intervention group achieved greater improvements in lipid profiles, with significant reductions in total cholesterol (*P*<.001), triglycerides (*P*<.001), and low-density lipoprotein cholesterol (*P*<.001), while high-density lipoprotein cholesterol remained stable (*P*=.45).

**Conclusions:**

This study demonstrates that a novel multidimensional digital CR program is acceptable and effective in improving functional status and health-related quality of life in patients with UA undergoing PCI within a short timeframe.

## Introduction

Secondary prevention of cardiovascular disease (CVD) is essential and effective in reducing recurrent cardiovascular events in patients with acute coronary syndrome (ACS); however, its global implementation remains suboptimal [1]. Notably, exercise-focused cardiac rehabilitation (CR) is a fundamental component of secondary prevention for CVD and holds a Class IA recommendation in European guidelines [1]. CR is crucial in restoring muscle strength, endurance, and cardiac function, enabling patients to resume daily activities and work. In addition, CR provides critical emotional and psychological support, which is essential for managing anxiety, depression, and stress associated with coronary heart disease (CHD) [2,3]. Despite its well-documented benefits, CR remains underused, with patient participation rates failing to meet expectations. Fewer than 50% of eligible patients with CHD participate in CR after an acute coronary event [4]. Key factors contributing to this issue include shortened hospital stays and insufficient rehabilitation guidance during hospitalization. Furthermore, fewer than 60% of recipients of percutaneous coronary intervention (PCI) demonstrate satisfactory compliance with CR programs [5]. Multiple factors, such as age, comorbidities, subjective fear, reduced exercise endurance, and impaired balance function, contribute to low participation rates, poor compliance, and suboptimal rehabilitation outcomes [6]. Therefore, addressing this challenge requires simple, cost-effective, and adaptable strategies to bridge the implementation gap.

Digital health technologies (DHTs) offer a noninvasive and emerging approach to disease intervention, aiming to improve patient lifestyle and prognosis [7-11]. A DHT-based, short-term personalized exercise rehabilitation program can enhance exercise capacity and health-related quality of life, functioning as an alternative or supplement to conventional face-to-face CR [12]. However, the effectiveness of integrating DHTs into CR for patients with unstable angina (UA) undergoing PCI remains underexplored. This prospective, emulated target trial evaluated the impact of a multidimensional digital CR package for patients with UA undergoing PCI.

## Methods

### Study Design and Population

This emulated target trial was conducted at the First Affiliated Hospital of Bengbu Medical University, enrolling 164 patients with UA who underwent PCI in the Department of Cardiology. The control group included patients from April to May 2022, while the intervention group consisted of those treated from May to June 2022. This study was conducted without any significant changes in nursing care or institutional protocols during the recruitment period. Both the control and intervention groups were treated under the same standard of care, and no variations in nursing practices or care delivery occurred between the 2 groups during their respective recruitment periods. Both groups visited the same study site and followed an identical trial protocol, with the intervention as the only distinction. The inclusion criteria were as follows: (1) patients diagnosed with UA who successfully underwent PCI; (2) patients classified as having cardiac function grades 1 to 3; (3) patients with no new or recurrent chest pain for at least 8 hours after reperfusion therapy, no new arrhythmias or electrocardiogram changes within this timeframe, and no further elevation in myocardial injury marker levels; (4) patients capable of using a smartphone; (5) patients who understood the purpose of the study and voluntarily provided written informed consent. The exclusion criteria included the following: (1) previous PCI surgery; (2) patients outside the age range of 18-74 years old; (3) patients with significant postoperative wound bleeding, severe infection, fever, severe electrolyte-acid-base imbalance, severe hypoxia, or malnutrition; (4) patients classified as having cardiac function grade 4; (5) patients with hepatic and renal failure; (6) patients with severe physical mobility impairments; (7) refusal to sign the consent form; (8) mental abnormalities; and (9) patients deemed unsuitable for participation.

The multidimensional digital CR intervention group and the usual care group were matched based on propensity scores calculated using logistic multiple regression analysis to minimize potential confounding factors. The matching process considered covariates such as age, sex, education level, marital status, exercise habits, monthly salary, current address, living arrangement (solitary or not), history of CHD, history of PCI, length of hospitalization, medical insurance status, and polypharmacy data obtained from presurveys. Propensity score analysis was performed using a 1-to-1 ratio matching approach with the nearest neighbor method to ensure balanced characteristics between the intervention and control groups. A standard caliper width of 0.2 was applied to the propensity score. After matching, 136 patients remained, with 68 in the multidimensional digital CR group and 68 in the usual care group.

### Study Procedures

Participants were assessed at baseline (upon admission) and again 3 months postsurgery. Standardized questionnaires collected baseline data from the multidimensional digital CR intervention and usual care groups during recruitment, including sociodemographic characteristics and medical history. At discharge, information on home medication use was recorded for both groups. During the 3-month follow-up, participants completed another round of standardized questionnaires and underwent assessments of lipid levels, quality of life, physical fitness, and frailty status. Patient characteristics and outcomes were obtained through medical record reviews by trained nurses, clinic visit evaluations, and participant self-reports. Additional details on data collection are available in Multimedia Appendix 1.

### Study Intervention

Following UA treatment guidelines, physicians provide standardized treatment to all patients and educate them on UA and PCI [13]. In the multidimensional digital CR intervention group, a nurse-led multidisciplinary team provided rehabilitation therapy through a customized digital CR program, which was implemented in 2 phases: during hospitalization and after discharge.

During hospitalization, nurses delivered a comprehensive and personalized multidimensional CR program to patients in the intervention group. This program encompassed 7 key rehabilitation components: exercise, medication management, nutritional guidance, psychological support, sleep management, health education, and smoking cessation assistance. Integrated into bedside tablet computers, the program provided multimedia content, including instructional videos, images, and written explanations, enabling patients to learn and follow their rehabilitation routines at their own pace. Nurses provided daily, one-on-one guidance in multimedia-equipped hospital rooms, ensuring that exercises were performed correctly, safely, and tailored to individual needs. The exercise regimen was customized based on each patient’s physical condition, focusing on progressive muscle strengthening, aerobic training, and flexibility. Exercise intensity was adjusted according to patients’ fitness levels, as assessed by their Borg score after a 6-minute walk test (6MWT), with noninvasive cardiac output monitoring used to ensure safety and efficacy. In addition to exercise training, the CR program included 2 weekly health guidance sessions, which covered essential topics such as medication management, nutritional guidance, psychological support, sleep management, and smoking cessation assistance. Nurses provided personalized education on the correct use of medications, including statins, aspirin, blood pressure medications, and glucose-lowering drugs. Educational videos featured health care professionals explaining the role of these medications in stabilizing coronary arteries and preventing further complications. In addition to medication education, patients received dietary advice focused on heart-healthy eating habits, such as reducing saturated fat intake, increasing fruits and vegetables, and limiting sodium and sugar. Nutritional guidance was personalized according to each patient’s health status and preferences, ensuring that dietary changes were both feasible and sustainable. Psychological support was another critical component of the CR program. Nurses conducted weekly sessions designed to address emotional well-being and stress management. These sessions included relaxation techniques, mindfulness exercises, and educational resources to help alleviate anxiety, depression, and fear related to recovery. By improving patients’ mental resilience, the program aimed to foster a more positive outlook and greater engagement in the rehabilitation process. Sleep management was also a key focus of the intervention. Nurses provided education on good sleep hygiene practices, such as establishing a regular sleep schedule, avoiding stimulants before bedtime, and creating an optimal sleep environment. For patients who reported sleep disturbances, personalized strategies were developed to improve sleep quality and promote better recovery outcomes. The program also offered smoking cessation assistance. Nurses provided behavioral counseling and motivational support, helping patients understand the harmful effects of smoking on heart health and guiding them through various strategies to quit, including nicotine replacement therapy. The intervention included regular follow-up to track progress and offer encouragement. Finally, nurses taught patients essential self-management skills, such as weight measurement, blood pressure monitoring, pulse checking, and blood glucose testing. The goal was to empower patients to actively participate in their recovery by equipping them with the tools necessary for managing their health after discharge. The detailed process of the CR program during hospitalization is outlined in Multimedia Appendix 1.

After discharge, one-on-one follow-up education was provided via WeChat software (Tencent) during the first, second, and third months postsurgery. Outpatient follow-ups were conducted in the third month after surgery. During these follow-ups, patients updated the multidisciplinary intervention team on their health status, including blood pressure, physical activity, eating habits, sleep patterns, psychological well-being, and smoking cessation progress. Nurses developed personalized rehabilitation plans based on each patient’s rehabilitation status. At discharge, nurses also trained all patients’ families to improve symptom recognition and management. All exercise rehabilitation program materials were converted into QR codes to facilitate patient self-learning after discharge.

The CR program package was entirely developed and guided by a multidisciplinary intervention team of cardiovascular nurses, cardiovascular clinicians, and rehabilitation physicians. This approach included essential information on disease diagnosis, treatment, self-management, and home rehabilitation. The multidisciplinary team systematically reviewed and summarized the best available evidence on exercise rehabilitation for patients with coronary artery disease [12] and designated an exercise program using Flash frame-by-frame animation technology, accommodating patients with varying physical capacities. The program incorporated simple, accessible language with corresponding videos, audio, and subtitles in standard Mandarin to reduce barriers to health education and rehabilitation guidance, including language and literacy challenges (Multimedia Appendix 2). Regular quality control and feedback mechanisms maintained the effectiveness of the CR program package. Detailed content of the CR program is provided in Multimedia Appendix 1.

Patients in the usual care group received standardized treatment, routine nursing care, and health education. Routine nursing care and health education were delivered through verbal instruction and the “317 Nursing Education Platform.” All patients underwent PCI treatment and completed a 3-month follow-up.

### Measurements

Physical fitness was evaluated using grip strength, gait speed, the 30-second chair stand test (30-s CST), BMI, and waist circumference (WC). Grip strength was measured with an electronic grip device (dominant hand, 3 attempts). The gait speed was assessed by timing participants as they walked a 4.6-m distance as quickly as possible using a stopwatch. The 30-s CST recorded the maximum number of times a participant could stand up and sit down within 30 seconds while maintaining a standard posture. BMI was calculated by dividing the body weight in kilograms by the square height in meters. WC was measured in centimeters at the midpoint between the lower rib and iliac crest while the participant stood relaxed, with measurements taken at the end of a normal exhalation. The cardiorespiratory function was assessed using the 6MWT, measuring the maximum distance within 6 minutes. Frailty was determined using the frailty phenotype (FP) scores based on weight loss, gait speed, grip strength, fatigue, and physical activity levels. Frailty was diagnosed if 3 or more indicators were met, while prefrailty was identified when 1 or 2 were present. Quality of life was measured using the 12-item Short Form Health Survey (SF-12), which evaluated physical and mental health. Physical and mental component summary scores were calculated, with higher scores reflecting better quality of life. Blood lipid levels, including total cholesterol (TC), high-density lipoprotein cholesterol (HDL-C), low-density lipoprotein cholesterol (LDL-C), and triglycerides (TG), were analyzed in the hospital laboratory. Details of all measurements and quality control procedures are available in Multimedia Appendix 1.

### Outcomes

All clinical outcome measurements were collected at the 3-month follow-up. Primary outcomes included changes in the 6MWT, SF-12, and FP scores. Secondary outcomes encompassed gait speed, 30-s CST, grip strength, WC, BMI, and alterations in lipid profiles (TC, HDL-C, LDL-C, and TG) at 3 months. Definitions of clinical outcomes and quality control details are provided in Multimedia Appendix 1.

### Randomization and Masking

Due to the nature of the intervention, random assignment of the participants was not feasible. The statistical analysts responsible for data analysis and researchers conducting patient outcome evaluations during follow-up remained blinded to minimize bias.

### Statistical Analysis

Statistical analysis was conducted using R software (version 4.2.0; The R Core Team). Continuous variables following a normal distribution are presented as the mean SD, with differences between groups assessed using *t* tests. Non-normally distributed continuous variables are expressed as median (IQR) values, and group comparisons were performed using the Wilcoxon test. Categorical variables are reported as frequencies (percentages), with group differences evaluated using either the chi-square test or Fisher exact probability method. A *P* value<.05 was considered statistically significant.

### Ethical Considerations

This study was reviewed and approved by the Ethics Committee of the First Affiliated Hospital of Bengbu Medical University (approval no. 2020KY100) and registered with the China Clinical Trials Register (ChiCTR2200059265). Written informed consent was obtained from all participants before enrollment. As this constitutes the inaugural investigation of the multidimensional digital CR intervention, the collected data are original and not derived from secondary analyses of preexisting datasets. All study data were anonymized and deidentified to safeguard participant confidentiality and privacy. Given that the intervention is noninvasive and noncompensatory in nature, no financial remuneration was provided to the participants. Furthermore, we confirm that no identifiable images of participants are included in either the manuscript or in Multimedia Appendix 1.

## Results

### Baseline Characteristics

**Figure 1.**
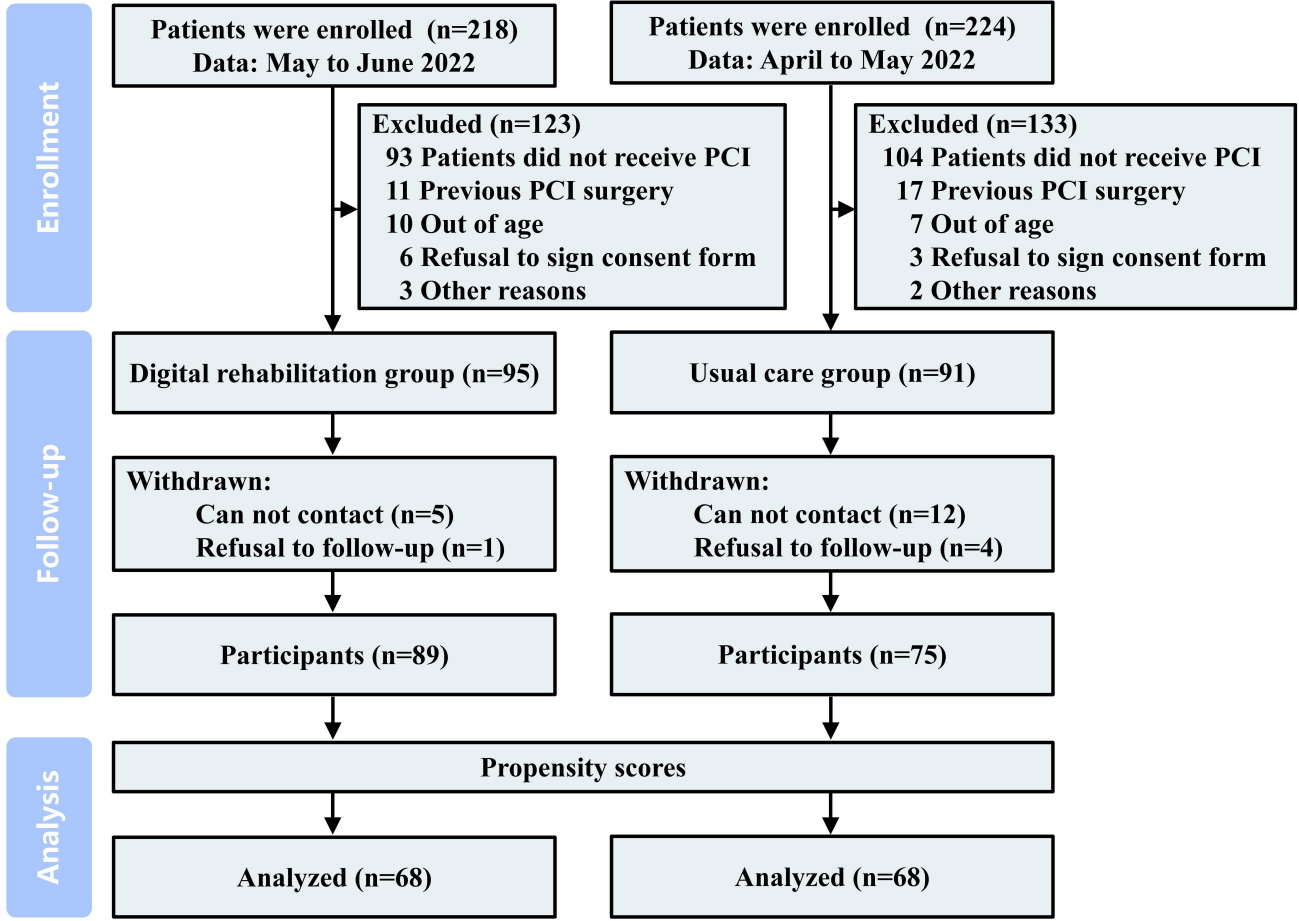
Screening, randomization, and follow-up flowchart. The study was conducted at the First Affiliated Hospital of Bengbu Medical University, April-June 2022, and enrolled 164 patients with unstable angina undergoing percutaneous coronary intervention. PCI: percutaneous coronary intervention.

Baseline characteristics were analyzed using propensity score matching. A total of 164 patients were included, with 89 assigned to the multidimensional digital CR intervention group and 75 to the usual care group. After applying a 1:1 matching approach to adjust for baseline inconsistencies, 68 patients were selected from each group (Figure 1). Statistically, nonsignificant differences were observed between the 2 groups after matching baseline data (*P*≥.05). Among 136 patients undergoing PCI (mean age: 70, SD 6.67 years), 59 (43.38%) were female. Table 1 provides an overview of the baseline characteristics.

**Table 1.** Baseline characteristics between the control group and the intervention group.

Characteristics	Total (N=136)	Intervention group (n=68)	Control group (n=68)	*P* value
Male, n (%)	59 (43.38)	29 (42.65)	30 (44.12)	>.99
Age (years), mean (range)	70 (6.55)	69 (6.42)	70 (6.67)	.29
Educational attainment, n (%)				.83
Less than high school	110 (80.88)	54 (79.41)	56 (82.35)	
High school and above	26 (19.12)	14 (20.59)	12 (17.65)	
Marital status, n (%)				.79
Single (unmarried, divorced, and widowed)	16 (11.76)	7 (10.29)	9 (13.24)	
Married	120 (88.24)	61 (89.71)	59 (86.76)	
Exercise habit, n (%)	31 (22.79)	15 (22.06)	16 (23.53)	>.99
Medical insurance status, n (%)				>.99
Employee medical insurance	41 (30.15)	21 (30.88)	20 (29.41)	
Residents’ medical insurance	95 (69.85)	47 (69.12)	48 (70.59)	
Monthly income (CNY^a^), n (%)				.49
<2000	79 (58.09)	37 (54.41)	42 (61.76)	
>2000	57 (41.91)	31 (45.59)	26 (38.24)	
Current address, n (%)				.73
City	71 (52.21)	37 (54.41)	34 (50)	
Countryside	65 (47.79)	31 (45.59)	34 (50)	
Solitary living arrangement, n (%)	10 (7.35)	4 (5.88)	6 (8.82)	.74
Duration of coronary heart disease (years), median (IQR)	2 (0‐6)	2 (0‐6.5)	1 (0‐6)	.50
History of PCI^b^, n (%)	49 (36.03)	23 (33.82)	26 (38.24)	.72
Smoking, n (%)	17 (12.5)	10 (14.7)	7 (10.3)	.44
Comorbidities, n (%)				
Hypertension	77 (56.6)	37 (54.4)	40 (58.8)	.60
Diabetes mellitus	42 (30.9)	22 (32.4)	20 (29.4)	.71
Stroke	50 (36.8)	28 (41.2)	22 (32.4)	.29
Number of drugs, median (IQR)	5 (4-5)	4 (4-5)	5 (4-5)	.66

a All monetary values in Chinese Yuan (CNY) were converted to US dollars using the official exchange rate published by the China Foreign Exchange Trade System on May 5, 2022: US $1=CNY ¥6.5672 (or CNY ¥1=US $0.1523).

b PCI: percutaneous coronary intervention.

### Frailty Status

**Figure 2.**
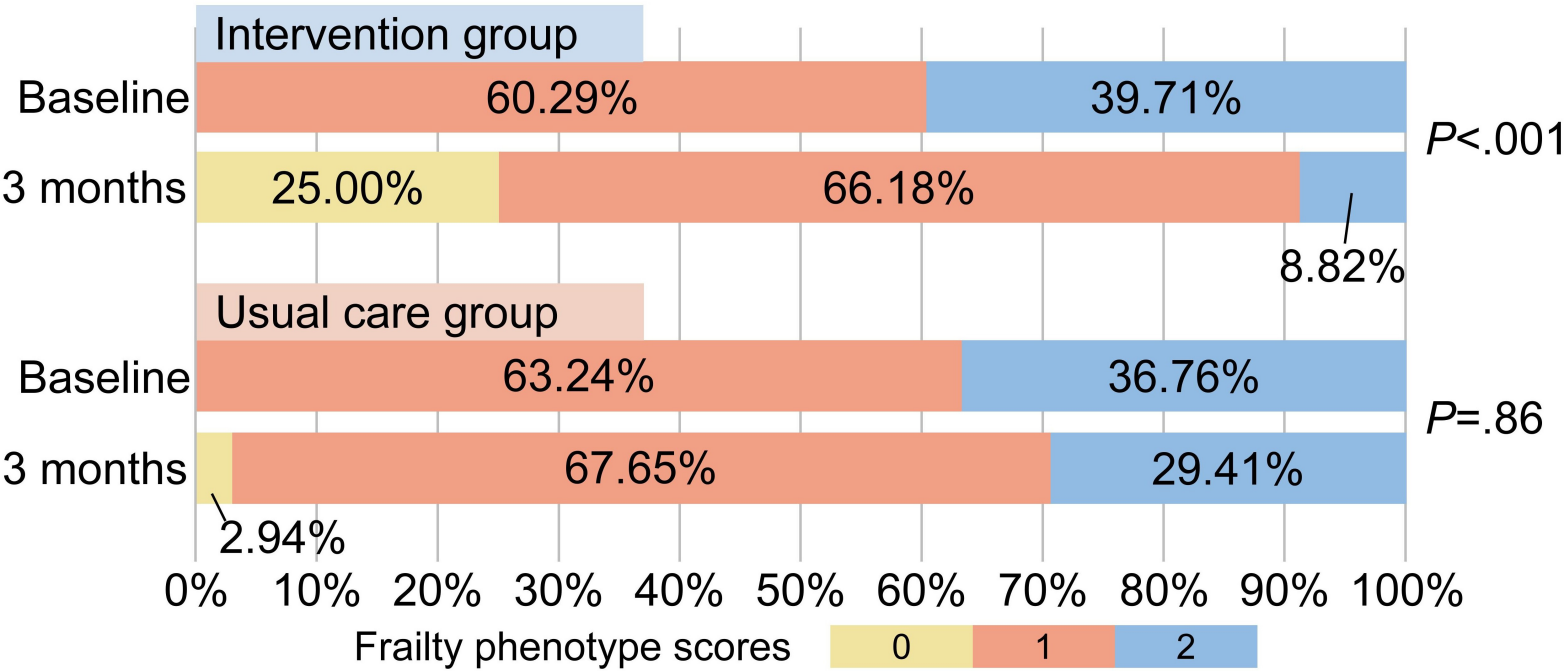
Comparison of frailty phenotype scores between the multidimensional digital cardiac rehabilitation intervention and usual care groups in patients with unstable angina undergoing percutaneous coronary intervention at baseline and the 3-month follow-up.

Figure 2 and Table S1 in Multimedia Appendix 3 indicate a significant improvement in the FP status within the intervention group at the 3-month follow-up compared to baseline. The proportion of prefrail patients declined from 100% (68 patients) to 75% (51 patients), while nonfrail patients increased from 0 to 17 (25%; *P*<.001). In the control group, the number of frail patients decreased from 68 (100%) to 66 (97.06%), and nonfrail patients increased from 0 to 2 (2.94%; *P*=.86). The intervention group exhibited a notably lower proportion of frail patients than the control group.

### Physical Fitness

**Figure 3.**
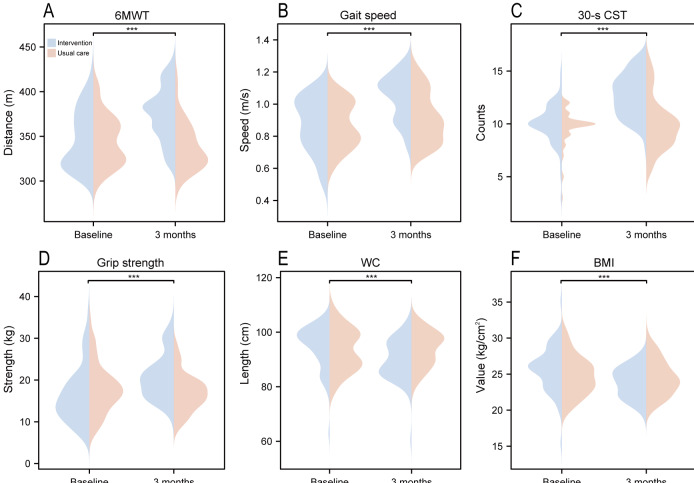
Comparison of the 6MWT, gait speed, 30-s CST, grip strength, BMI, and waist circumference between the multidimensional digital cardiac rehabilitation intervention and the usual care groups in patients with unstable angina undergoing percutaneous coronary intervention at baseline and the 3-month follow-up. 6MWT: 6-minute walk test; 30-s CST: 30-second chair stand test; WC: waist circumference. ***P<.001.

Figure 3 and Table S2 in Multimedia Appendix 3 present a comparison of physical fitness between the 2 groups, revealing significant differences over time in the 6MWT, gait speed, and 30-s CST. Time, group, and interaction effects reached statistical significance (all *P*<.001, except for gait speed, where *P*=.02 for the group effect). The intervention group demonstrated improvements in 6MWT (347.06, SD 32.43 to 375.22, SD 29.71 m), gait speed (0.87‐1.04 m/s), and 30-s CST (10.0, SD 1.89 to 12.71, SD 1.97). In contrast, the control group experienced a decrease in 6MWT (345.15, SD 27.76 to 338.15, SD 26.47 m), a slight increase in gait speed (0.87‐0.91 m/s), and a smaller increase in 30-s CST (9.76, SD 1.63 to 10.0, SD 2.42). Significant main effects of time and time-group interactions were observed for grip strength, BMI, and WC (all *P*<.001). In the intervention group, grip strength increased from 16.64 (SD 6.57) to 20.74 (SD 5.37), BMI declined from 25.74 (SD 3.05) to 23.88 (SD 2.14), and WC reduced from 94.98 (SD 7.87) to 89.91 (SD 7.50). Conversely, in the control group, grip strength declined from 17.81 (SD 5.80) to 17.01 (SD 4.51), BMI exhibited a slight reduction from 24.77 (SD 2.70) to 24.49 (SD 2.48), and WC increased from 93.49 (SD 7.69) to 93.93 (SD 6.40). These findings indicate that the intervention group achieved greater improvements in grip strength, WC, and BMI over time compared to the control group.

### Quality of Life

**Figure 4.**
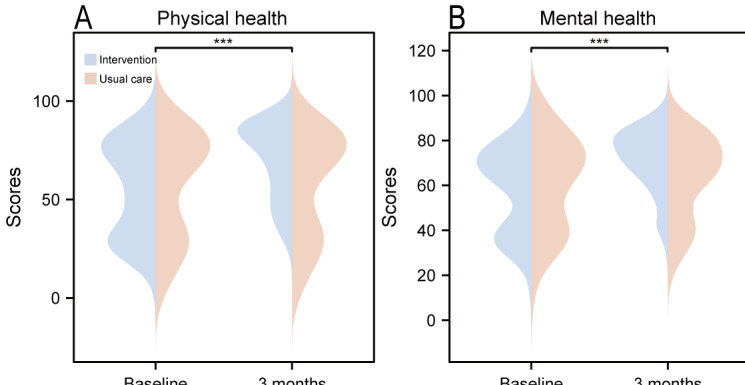
Comparison of 12-item Short Form Health Survey (SF-12) scores between the multidimensional digital cardiac rehabilitation intervention and the usual care groups in patients with unstable angina undergoing percutaneous coronary intervention at baseline and 3-month follow-up. ***P<.001.

Figure 4 and Table S3 in Multimedia Appendix 3 present changes in SF-12 scores between the 2 groups. In the intervention group, the physical health score increased from 55.11 (SD 23.53) to 70.31 (SD 18.83), while the mental health score rose from 58.48 (SD 18.59) to 71.68 (SD 13.78). In contrast, the control group exhibited minimal changes, with the physical health score increasing from 57.22 (SD 26.86) to 57.45 (SD 26.63) and the mental health score rising from 60.85 (SD 21.14) to 63.44 (SD 18.33). The main effect of time and the time-group interaction was statistically significant for both physical health and mental health (*P*<.001). Conversely, the main effect of the group demonstrated a statistically nonsignificant difference (*P*=.19 for physical health, *P*=.39 for mental health).

### Lipid Levels

**Figure 5.**
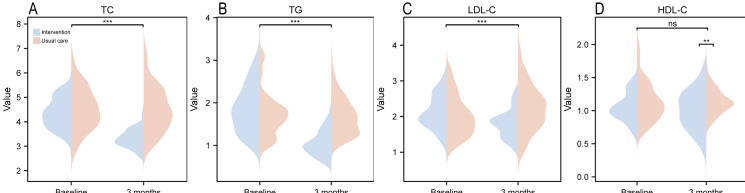
Comparison of total cholesterol (TC), triglyceride (TG), and low-density lipoprotein cholesterol (LDL-C) and high-density lipoprotein cholesterol (HDL-C) between the multidimensional digital cardiac rehabilitation intervention and the usual care groups in patients with unstable angina undergoing percutaneous coronary intervention at baseline and the 3-month follow-up. ***P<.001; **P<.01; ns: *P*>.05.

Figure 5 and Table S4 in Multimedia Appendix 3 illustrate changes in lipid profiles between the intervention and control groups. In the intervention group, TC decreased from 4.34 (SD 0.59) mmol/L at baseline to 3.37 (SD 0.37) mmol/L at 3 months, TG declined from 1.86 (SD 0.55) to 1.04 (SD 0.28) mmol/L, and LDL-C reduced from 2.13 (SD 0.46) to 1.80 (SD 0.39) mmol/L. HDL-C remained stable, shifting slightly from 1.05 (SD 0.21) to 1.01 (SD 0.29) mmol/L. In the control group, TC decreased from 4.51 (SD 0.86) to 4.51 (SD 0.91) mmol/L, TG declined from 1.71 (SD 0.51) to 1.68 (SD 0.43) mmol/L, LDL-C increased from 2.01 (SD 0.55) to 2.23 (SD 0.64) mmol/L, and HDL-C remained stable at 1.13 (SD 0.29) mmol/L at baseline and 1.14 (SD 0.18) mmol/L at 3 months. The main time effect and the time-group interaction were statistically significant for TC and TG (*P*<.001), while the main time effect exhibited a statistically nonsignificant difference (*P*=.51 for LDL-C). Furthermore, the main group effect for HDL-C was statistically significant (*P*=.002).

## Discussion

### Principal Findings

**Figure 6.**
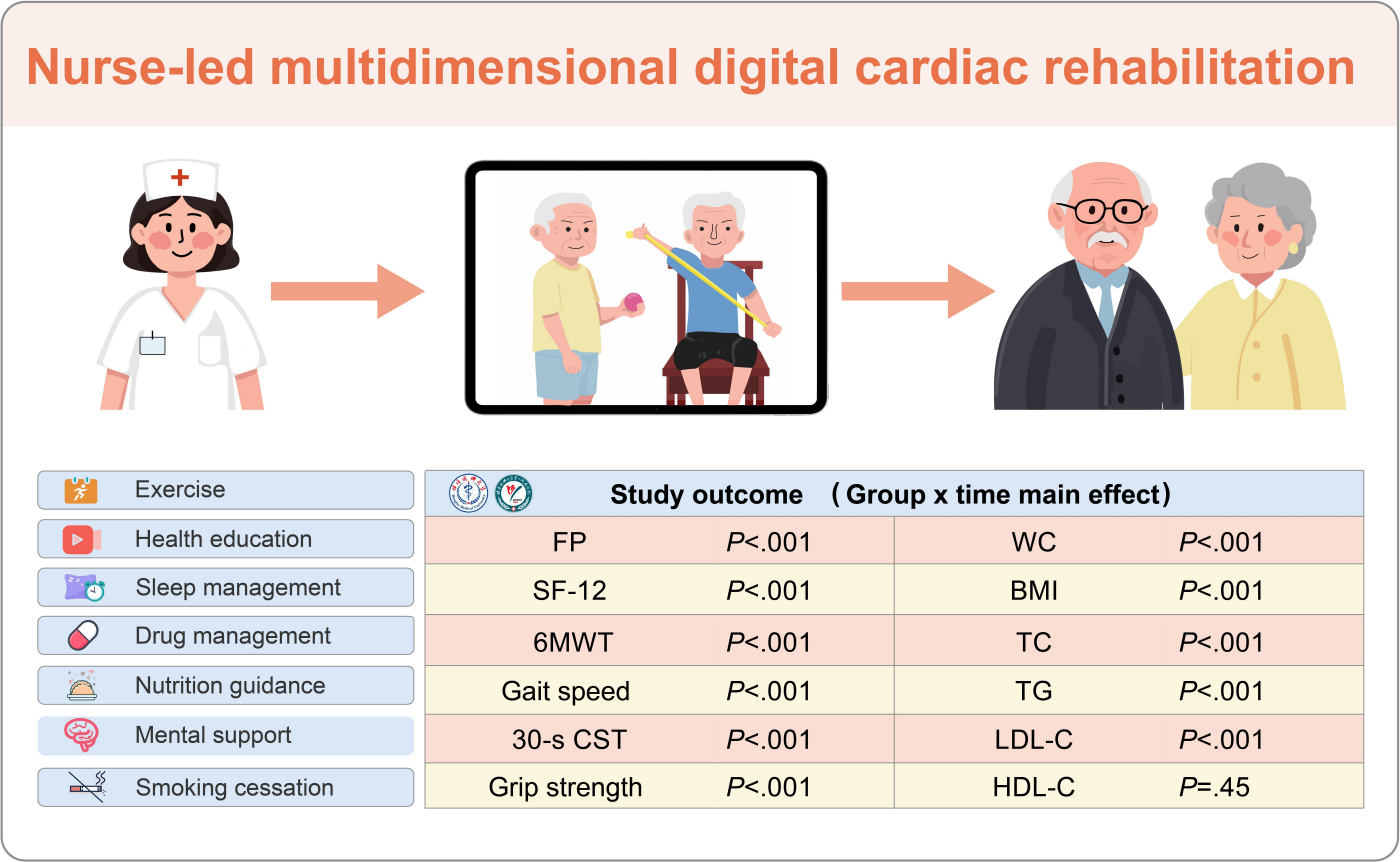
Visual summary of nurse-led multidimensional digital cardiac rehabilitation. FP: frailty phenotype; SF-12: 12-item Short Form Health Survey; 6MWT: 6-minute walk test; 30-s CST: 30-second chair stand test; WC: waist circumference; TC: total cholesterol; TG: triglyceride; HDL: high-density lipoprotein cholesterol; LDL-C: low-density lipoprotein cholesterol.

Findings indicated that a multidimensional digital CR intervention improved functional status (FP, 6MWT, gait speed, and 30-s CST) and metabolic markers (TC, TG, and LDL-C) in patients with UA undergoing PCI within a relatively short period compared to standard management. These results emphasize the program’s potential to enhance physical recovery and cardiovascular health. Significant time effects were also observed for grip strength, WC, BMI, and overall quality of life (physical and mental components), reinforcing the efficacy of digital interventions. This novel rehabilitation model provides CVD education, clarifies essential diagnostic procedures, provides exercise rehabilitation guidance, and supports home-based rehabilitation. Promoting physical activity, encouraging lifestyle modifications, and facilitating behavioral changes during recovery contribute to improved quality of life in patients with UA undergoing PCI (Figure 6).

### Comparison With Existing Research

In clinical practice, PCI and long-term lipid-lowering treatment are essential strategies for enhancing long-term prognosis in patients with UA. Despite these efforts, the long-term prognosis remains suboptimal for some individuals [14]. Contributing factors may include inadequate physical activity, persistence of risk factors, or low medication adherence, all of which directly influence the effectiveness of PCI and overall quality of life [15-17]. Rehabilitation training plays a critical role in mitigating these adverse outcomes. CR is a comprehensive intervention encompassing exercise training, medication adherence, lifestyle changes, educational support, and psychosocial assessments [3]. Multiple guidelines advocate exercise-based CR as a key component of secondary prevention for ACS, demonstrating significant benefits in prognosis and a reduced risk of major adverse cardiovascular events [18]. A recent multicenter randomized controlled trial evaluated the clinical benefits of early, individualized, low-cost exercise interventions for older patients hospitalized with ACS [19]. Findings demonstrated that exercise interventions greatly improved patients’ quality of life, reduced anxiety and depression, and lowered rates of cardiac-related mortality and rehospitalization. Despite the well-established efficacy and safety of CR, participation rates among patients with ACS remain low, and compliance is poor. DHTs have emerged as a valuable adjunct, offering new perspectives in CR. A mixed method study [20] found that virtual CR yielded comparable improvements in functional performance to traditional face-to-face CR while being more accepted by patients and health care providers. Research by Xu et al [21] indicates that remote health interventions effectively enhance exercise adherence, planning, engagement, and cognitive levels in patients with CHD undergoing CR. These findings suggest that DHTs strengthen traditional CR by improving comprehension, acceptance, and implementation, adding importance to conventional rehabilitation programs [22-24]. However, sustaining healthy lifestyle changes remains challenging, and systematic evaluation is difficult. Consequently, designing a more comprehensive, cost-effective, and targeted digital CR intervention is essential to address the clinical characteristics and personalized rehabilitation needs of this population.

Numerous studies emphasized the critical role of exercise rehabilitation in cardiac recovery, demonstrating the significant effects of various exercise modalities on rehabilitation outcomes [25]. A randomized trial examined the impact of Yoga-caRe on improving self-rated health and facilitating a return to preinfarct activities, highlighting yoga-based rehabilitation as a viable option for patients when conventional CR programs are unavailable or poorly received [26]. Another randomized controlled trial found that a 6-month Tai Chi program provided a safe and effective alternative to traditional CR, significantly enhancing physical activity levels, weight management, and quality of life in patients with CHD who are reluctant to engage in conventional rehabilitation programs [27]. These findings underscore the importance of selecting appropriate exercise modalities for optimal CR. In contrast to previous studies, this study customized exercise interventions specifically for patients with UA undergoing PCI, incorporating considerations such as age and accessibility. Based on the “Baduanjin” exercise [28,29], movements were refined to accommodate patients with varying physical capabilities. While some studies on digital CR have demonstrated significant benefits in diverse patient populations with ACS, research on patients undergoing PCI, particularly those with UA, remains limited.

This study developed a targeted multidimensional digital CR program for patients with UA undergoing PCI. The program comprises 7 key dimensions: exercise, medication management, nutritional guidance, psychological support, sleep management, health education, and smoking cessation assistance. These interventions aim to reduce patients’ frailty, improve physical function, optimize lipid levels, and enhance mental well-being and quality of life. Furthermore, the rehabilitation program integrates medication management, nutritional guidance, psychological support, sleep management, health education, and smoking cessation assistance, all digitized via QR codes on the WeChat platform for a comprehensive multidimensional approach. A personalized exercise program was designed using Flash frame-by-frame animation technology to accommodate varying fitness levels [30]. In addition to exercise interventions, psychological health, medication adherence, and risk factor management remain integral components of CR [31].

The importance of prehabilitation training and family support is increasingly recognized [32]. A meta-analysis demonstrated that isolated exercise and nutritional interventions, as well as combined approaches, significantly reduce postoperative complications, shorten hospital stays, and improve health-related quality of life [33]. In addition, a randomized controlled trial examined the effects of incorporating basic life support training into a CR program on the skills and attitudes of relatives of patients with ACS. The findings revealed that relatives in the resuscitation-retraining program retained basic life support skills significantly better and reported higher self-perceived preparedness after 6 months than those in the standard program [34]. Consequently, patients gained early exposure to standardized rehabilitation content. The same rehabilitation training program was also provided to family members, equipping them to offer support and reinforce the benefits of health education at home [34]. These interventions collectively improved patients’ frailty and physical function, optimized lipid profiles, and enhanced mental well-being and overall quality of life.

### Limitations

This study has some limitations. First, it was conducted at a single center in China with a patient population predominantly composed of rural residents with low educational levels, potentially limiting the generalizability of the findings to younger, more urban, or more highly educated populations. Second, as a nonrandomized clinical trial with a before-and-after control design, the possibility of maturation bias cannot be entirely excluded despite efforts to match control participants and blind outcome assessors. Third, the short follow-up period did not provide insights into the long-term stability of lipid metabolism indicators or the recurrence rate of cardiovascular events. Consequently, further research with extended follow-up is essential to fully assess the sustained impact of the intervention on these critical outcomes.

### Conclusions

Findings indicate that a multidimensional digital CR intervention can effectively complement standard care by improving key health outcomes in patients with UA. This approach enhances patient engagement and adherence to rehabilitation programs through personalized and continuous support throughout recovery. Health care providers should consider integrating DHTs into CR programs to overcome patient participation barriers and optimize recovery and survival outcomes.

## Supplementary material

10.2196/75325 Multimedia Appendix 1 Trial protocol and analysis plan.

10.2196/75325 Multimedia Appendix 2 Screenshot from the exercise rehabilitation video in the multidimensional digital cardiac rehabilitation program.

10.2196/75325 Multimedia Appendix 3 Supplementary data analysis, including Tables S1-S4.

## Abbreviations

30-s CST: 30-second chair stand test
6MWT: 6-minute walk test
ACS: acute coronary syndrome
CHD: coronary heart disease
CR: cardiac rehabilitation
CVD: cardiovascular disease
DHT: digital health technology
FP: frailty phenotype
HDL-C: high-density lipoprotein cholesterol
LDL-C: low-density lipoprotein cholesterol
PCI: percutaneous coronary intervention
SF-12: 12-item Short Form Health Survey
TC: total cholesterol
TG: triglyceride
UA: unstable angina
WC: waist circumference
