# Supplementary material for: The Effectiveness of Nurse-Led Multidimensional Digital Cardiac Rehabilitation in Patients With Unstable Angina Undergoing Percutaneous Coronary Intervention: Emulated Target Trial
Source: J Med Internet Res. 2025 Aug 27;27:e75325. doi: 10.2196/75325 (PMC12384693; doi:10.2196/75325)
Supplement: Multimedia Appendix 1 [file jmir-v27-e75325-s001.pdf]

# Effectiveness of Nurse-led Multidimensional Digital Cardiac Rehabilitation in Patients with Unstable Angina Undergoing Percutaneous Coronary Intervention

# Trial Protocol and Statistical Analysis Plan

**Brief Title:** Benefits of Digital Multistage Cardiac Rehabilitation

**Principal Investigator**：Tongzhou

**Study Center:** Department of Cardiology, Renmin hospital of Wuhan University

**Address for correspondence:** No. 287, Changhuai Road, Longzihu District, Bengbu City, Anhui Province, China

**E-mail:** 13955217648@163.com

**Phone Number:** +86 13955217648

**Study Number:** No. 2020KY100

**Trial Registration:** No. ChiCTR2200059265

**Funding Support:** This work was supported by Clinical research transformation project of Anhui Province (202427b10020086, 202427b10020089, 202427b10020097), Research Funds of Joint Research Center for Regional Diseases of IHM (2024bydjk001, 2024bydjk002, 2024bydjk005) and Key Research Projects in Higher Education Institutions of Anhui Province (2023AH051901) .

**Original Protocol:** Version 1.0 November 2020

## Introduction

Unstable Angina (UA) is a critical subtype of Acute Coronary Syndrome (ACS) with high incidence and disability rates globally. In China, approximately 2.5 million new ACS cases are reported annually, with over 40% being UA, a figure expected to rise due to aging demographics and lifestyle changes. Although Percutaneous Coronary Intervention (PCI) effectively alleviates ischemic symptoms in UA patients, issues such as restenosis, recurrent cardiovascular events, and poor long-term prognosis remain significant. Studies show that the incidence of major adverse cardiovascular events (MACEs) within the first year post-PCI can reach 10%-15%, closely linked to patients' heart function, lifestyle, and adherence to rehabilitation management. Therefore, improving the long-term outcomes of UA patients post-PCI through effective cardiac rehabilitation (CR) interventions has become a vital aspect of secondary prevention in cardiovascular disease.

Traditional CR programs, encompassing exercise, risk factor management, and psychological support, have proven effective in reducing cardiovascular mortality and improving life quality. However, challenges such as low participation rates (<30%), regional disparities, and difficulties in long-term follow-up limit their widespread application. Recently, mobile health technologies (mHealth)-based remote CR have gained popularity, offering personalized, real-time guidance via smartphones, wearables, and remote monitoring systems. Initial studies suggest mHealth CR can improve physical endurance (e.g., a 15%-20% increase in 6-minute walk test) and reduce 30-day readmission rates. However, its efficacy, long-term effects, and safety in UA patients post-PCI remain underexplored.

This study aims to assess the effectiveness of a multidimensional mHealth-based cardiac rehabilitation program for UA patients undergoing PCI. Specific objectives include (1) comparing quality of life scores between the mHealth intervention and usual care groups at 6 months post-PCI, (2) exploring the impact of mHealth on patient adherence, self-management, and psychological well-being, and (3) providing evidence-based recommendations to optimize post-PCI rehabilitation strategies for UA patients and promote the clinical application of digital health technologies in

cardiovascular rehabilitation.

# Methods

## Study Rationale

The multidimensional digital cardiac rehabilitation intervention package used in this study was developed by the Department of Cardiology at the First Affiliated Hospital of Bengbu MedicalUniversity, specifically designed for the rehabilitation of patients with unstable angina (UA) who have undergone percutaneous coronary intervention (PCI). This intervention package is based on mHealth technology, offering the following advantages: it facilitates remote, personalized health management through a digital platform, overcoming the temporal and spatial limitations typically encountered in traditional cardiac rehabilitation. mHealth technology provides convenient remote guidance, ensuring that patients receive continuous and individualized support throughout their rehabilitation process. Particularly in terms of visualization, mHealth technology utilizes videos, images, and animations, helping patients intuitively understand rehabilitation content, accurately master exercise techniques, and treatment plans, thereby enhancing adherence and reducing the risk of errors. Through this interactive educational model, patients are better able to engage in the rehabilitation process, promoting improved treatment outcomes.

The intervention package integrates seven core dimensions: exercise training, medication management, nutrition guidance, psychological support, sleep management, health education, and smoking cessation support, aiming to provide patients with a comprehensive and personalized rehabilitation plan. All content was collaboratively developed by cardiologists, rehabilitation physicians, and nurses to ensure the professional quality and clinical feasibility of the program. The intervention package is delivered through a digital platform in various formats, including videos, images, and animations, enabling patients to access rehabilitation guidance and support through mobile devices during hospitalization and after discharge. We employed frame-by-frame animation technology to design exercise programs, ensuring that each patient's physical capacity and needs are addressed, thus

improving exercise adherence and avoiding the risks of overtraining.

The advantage of the multidimensional digital cardiac rehabilitation intervention package lies in its ability to provide personalized interventions tailored to each patient's specific condition, encompassing multiple aspects such as exercise, medication management, and nutrition guidance. The exercise training design not only helps improve cardiovascular function but also enhances muscle endurance, restoring the patient's ability to perform daily activities. The medication management dimension ensures that patients take their medications correctly and on time, reducing the occurrence of adverse drug reactions. Nutrition guidance assists patients in managing their weight and lowering blood lipids through appropriate dietary interventions, thus reducing the risk of cardiovascular disease recurrence. Psychological support, sleep management, and smoking cessation support help patients overcome the psychological stress caused by illness, improve sleep quality, and eliminate unhealthy lifestyle habits. These integrated interventions effectively improve the patient's overall health and contribute to the long-term maintenance of cardiovascular health.

Furthermore, this study places particular emphasis on the implementation of prehabilitation training, starting during hospitalization, which allows patients to become familiar with standardized rehabilitation content early on. During this process, the patients' family members were also included in the rehabilitation training program, ensuring they can provide essential support and enhance the role of health education within the home environment. These interventions collectively contributed to improvements in the patients' physical function, frailty, and lipid levels, while also enhancing their mental health and quality of life.

From a clinical application perspective, the digital rehabilitation package not only overcomes the temporal and spatial limitations of traditional inpatient treatment but also provides continuous and personalized rehabilitation support. Even after discharge, patients can continue to receive rehabilitation education, engage in self-management, and stay in contact with the medical team through the digital platform. This model is particularly beneficial for patients living in areas with limited access to medical

resources or those unable to attend regular outpatient treatments due to work or lifestyle factors. Moreover, the digital rehabilitation package enables real-time monitoring of the patient's rehabilitation progress, facilitating timely adjustments to treatment plans and ensuring optimal rehabilitation outcomes. Through this digital intervention model, we can significantly improve patient adherence to rehabilitation and treatment outcomes, providing a feasible practical solution for the future of digital health management.

**Study Purpose**

***Primary Objective:*** To assess improvement in 6-minute walk test (6MWT), 12-item Short Form Health Survey (SF-12) sores, and frailty phenotypic sores in UA patients undergoing PCI at 3 months postoperatively.

***Secondary Objectives:*** To evaluate the changes in gait speed, 30-second chair stand test (30-s CST), grip strength, waist circumference (WC), body mass index (BMI), total cholesterol (TC), high-density lipoprotein cholesterol (HDL-C) levels, low-density lipoprotein cholesterol (LDL-C) levels, and triglycerides(TG) levels were assessed three months after PCI in patients with UA.

**Study Design**

In this prospective, emulated target trial, we aim to evaluate the effectiveness of multidimensional digital cardiac rehabilitation in UA Patients undergoing PCI. In this study, the process of patient recruitment began when patients were diagnosed with unstable angina (UA), which was implemented in the First Affiliated Hospital of Bengbu Medical University. Potential participants were identified from the hospital's PCI registry, which included all patients with ACS requiring PCI. During this process, the research team conducted a detailed pre-screening process to ensure that patients met the inclusion criteria. The pre-screening criteria included: patients should be diagnosed with unstable angina and planned to undergo percutaneous coronary intervention (PCI); the patient's age, the stability of their condition and their ability to actively cooperate with rehabilitation training and related interventions were also used as part of the screening criteria. Patients who meet these criteria will be enrolled in the study to receive further health assessment and individualized interventions.

The research team recruits patients at the time of admission through the hospital's multimedia ward. The recruitment process is handled by a research team comprised of healthcare professionals from both the cardiovascular and rehabilitation departments, including attending physicians, nurses, rehabilitators, and research assistants, who provide patients with a detailed introduction to the study and an explanation of the intervention protocol during admission.

To ensure that patients understand the content of the study and the requirements for participation, the hospital is specially equipped with multimedia equipment, including video, graphic and animation displays, to help patients visualize the purpose of the study, the intervention protocol and the various study requirements. Through this multimedia ward environment, patients can receive comprehensive information about the study in a relaxed atmosphere, thus increasing their acceptance of the study and willingness to participate.

In addition, the research team has prepared relevant study materials and informed consent forms in advance to ensure that each patient is fully aware of his/her participation and related rights and interests before participating in the study. The research team will ensure that patients have signed the informed consent form before entering the study and will provide ongoing support and guidance throughout the study.

After the recruitment and pre-screening processes, patients were divided into two groups based on their admission dates: the control group (April to May 2022) and the intervention group (May to June 2022). In addition to receiving standardized treatment, the intervention group also received a multidimensional digital cardiac rehabilitation intervention, which encompassed seven dimensions: exercise training, medication management, nutritional guidance, psychological support, sleep management, health education, and smoking cessation support. It is important to note that, in addition to the intervention for patients themselves, the digital cardiac rehabilitation intervention particularly emphasized the involvement of the patients' family members. During the hospitalization period, family members were invited to participate in the rehabilitation training program, ensuring that they could provide

necessary support for the patients and promote health education within the home environment. The control group, on the other hand, received only standardized treatment and standard nursing care.

After the PCI procedure, both groups of patients underwent a three-month follow-up period. The follow-up included individualized educational sessions conducted monthly via the WeChat platform, in addition to outpatient visits at one and three months post-surgery. During the follow-up, patients were required to report their health status to the multidisciplinary intervention team, including blood pressure, physical activity, dietary habits, sleep patterns, psychological state, and progress in smoking cessation.

Only patients who completed all data collection requirements were included in the final analysis. The final dataset was subjected to propensity score matching to balance the baseline characteristics between the two groups, ensuring comparability.

**Study Duration**

Participants were asked to receive the intervention throughout their hospitalization and during post-discharge home rehabilitation and were followed for 3 months.

**Study Population**

This emulated target trial was conducted at the First Affiliated Hospital of Bengbu Medical University, including 164 UA patients who underwent PCI in the Department of Cardiology from April to May 2022 (the control group) and May to June 2022 (the intervention group). Main inclusion criteria were as follows: (1) Patients diagnosed with UA who successfully underwent PCI; (2) patients classified as having cardiac function grades 1 to 3; (3) patients with no new or recurrent chest pain for at least eight hours after reperfusion therapy, no new arrhythmias or ECG changes within this timeframe, and no further elevation in myocardial injury marker levels; (4) patients capable of using a smartphone; and (5) patients who understood the purpose of this study and voluntarily provided written informed consent. The exclusion criteria were as follows: (1) patients with significant postoperative wound bleeding, severe infection, fever, severe electrolyte–acid–base imbalance, severe

hypoxia, or malnutrition; and (2) patients who were unwilling to participate in this study or unable to adhere to the follow-up visit requirements.

***Inclusion and exclusion criteria.***

| Inclusion criteria |
| --- |
| Age between 18 and 74 years old |
| Patients diagnosed with UA who successfully underwent PCI |
| Patients classified as having cardiac function grades 1 to 3 |
| Patients with no new or recurrent chest pain for at least eight hours after reperfusion therapy, no new arrhythmias or ECG changes within this timeframe, and no further elevation in myocardial injury marker levels |
| Patients capable of using a smartphone |
| Patients who understood the purpose of this study and voluntarily provided written informed consent |
| Exclusion criteria |
| Previous PCI surgery |
| Out of age |
| Patients with significant postoperative wound bleeding, severe infection, fever, severe electrolyte–acid–base imbalance, severe hypoxia, or malnutrition |
| Patients classified as having cardiac function grades 4 |
| Patients with hepatic and renal failure |
| Patients with severe physical mobility impairments |
| Refusal to sign consent form |
| Mental abnormality |
| Not suitable for participation |

***Early termination of the study***

This study may be temporarily suspended or terminated early if there is good and reasonable cause for such termination.

| **Suspended or terminated criteria** |
| --- |
| Determination of unexpected, significant or unacceptable risks to participants |

| |
|---|
| Identify patients who may be at risk for an Serious Adverse Events(SAE) |
| Inadequate compliance with protocol requirements by the investigator |

**Interim analyses**

No interim analyses were planned or conducted.

**Screening, Randomization, and Follow-up Flowchart**

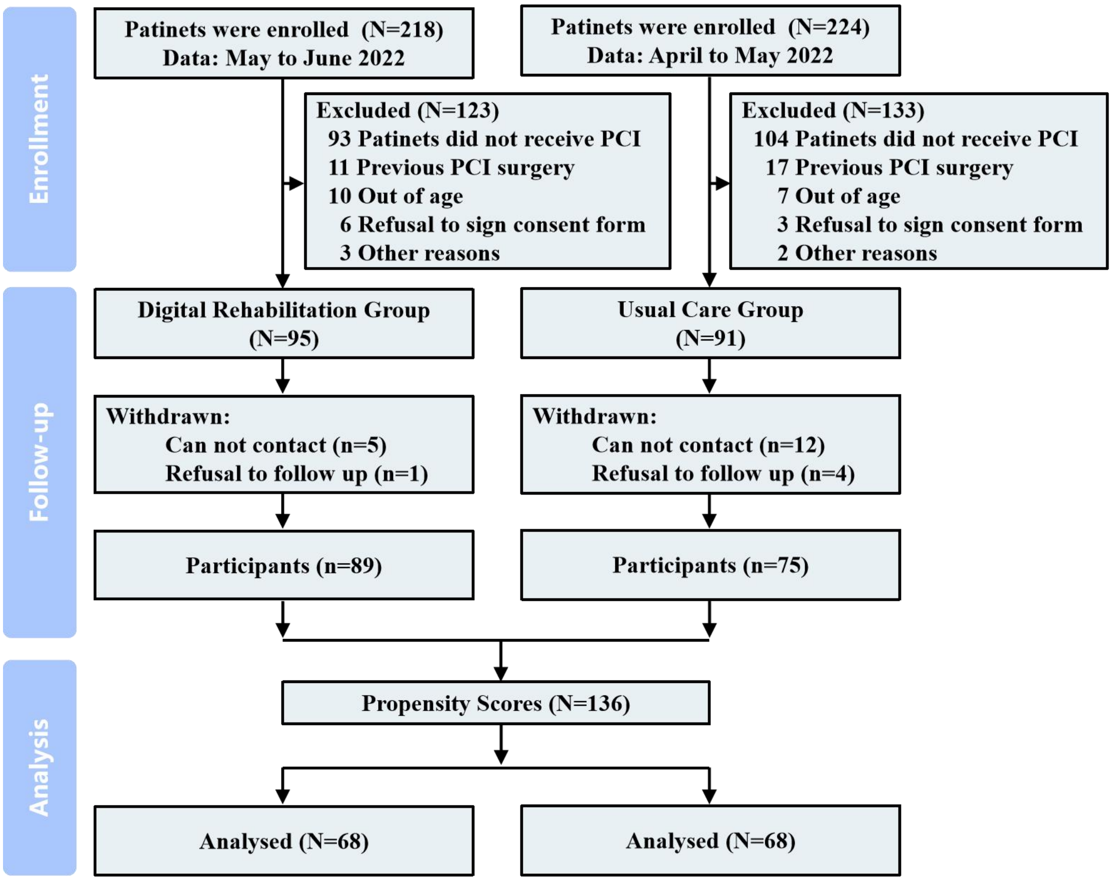


## Study Procedures

***Intervention Strategy***

According to the UA treatment guidelines, standardized treatment is provided to all patients, alongside essential knowledge regarding UA and PCI. In the multidimensional digital cardiac rehabilitation intervention group, patients received rehabilitation therapy managed by a multidisciplinary intervention team led by nurses. In this intervention teams, the Cardiovascular Medicine Nurse Manager was the primary person responsible for management of the project. One nursing evidence-based specialist screened expert panellists; four cardiovascular clinicians

and two rehabilitation physicians were involved in the design and implementation of the program; and 2 postgraduate students, consisting of stakeholders on a multidisciplinary team, were involved in the collection of statistical data and analysis of the data.

The multidimensional digital cardiac rehabilitation intervention was delivered through a customized digital cardiac rehabilitation program. The program includes seven key dimensions of rehabilitation: exercise, medication management, nutritional guidance, psychological support, sleep management, health education, and smoking cessation assistance.

***1. In-hospital phase***

The primary objective was to prepare patients for long-term recovery by providing education, personalized intervention, and comprehensive support.

***1.1 Exercise Training***

Exercise rehabilitation is a critical component of recovery following PCI, and the intervention group received personalized exercise plans designed to enhance cardiovascular health and physical capacity. During hospitalization, nurses delivered the exercise programs using multimedia-equipped bedside tablet computers, incorporating a variety of interactive materials such as instructional videos, images, and written explanations to ensure patients performed exercises correctly. Nurses provided one-on-one guidance during twice-weekly sessions, with a particular focus on progressive muscle strengthening, aerobic training, and flexibility. To accommodate varying physical capabilities, the exercise program was developed using Flash frame-by-frame animation technology, which allowed for clear visualization of movements, making it easier for patients to follow and execute exercises accurately.

Unlike previous studies, this research tailored the exercise interventions specifically for UA patients undergoing PCI, ensuring the exercises were suitable for individual fitness levels. The program incorporated "Baduanjin" exercises, optimizing movements based on patient age and physical condition, and ensuring accessibility for a diverse range of patients. Each participant received a written rehabilitation

prescription, which included a warm-up phase, aerobic training, strength training, balance exercises, and a cool-down period. The intensity of exercises was adjusted based on the patient's Borg score following a 6-minute walking test, combined with noninvasive cardiac output monitoring to ensure safety and effectiveness.

This individualized rehabilitation program was integrated into the bedside tablet computers, allowing patients to learn and replicate exercises through interactive content. Nurses reinforced the proper execution of the program, ensuring that the rehabilitation process was followed correctly and effectively. The overall goal was to provide a comprehensive, personalized exercise regimen that supported recovery, enhanced physical capacity, and catered to the diverse needs of the patient population.

### *1.2. Medication Management*

Effective medication management is essential for improving outcomes following PCI, and in the intervention group, nurses provided comprehensive medication education to ensure that patients understood the importance of their prescribed treatments. This education covered the correct timing, dosage, and potential side effects of medications, along with guidance on how to manage and adjust medications based on specific health indicators such as blood pressure and blood glucose levels. Nurses held one-on-one sessions twice a week to reinforce medication adherence, clarify any misconceptions, and ensure patients fully understood their treatment regimens.

In addition to medication education, patients were informed about behaviors that could help maintain their health and support recovery. Videos featuring healthcare practitioners were used to explain the role of medications like statins, aspirin, and Tegretol in stabilizing coronary arteries, alongside information about other common medications such as angiotensin-converting enzyme inhibitors and receptor blockers. Educational content also included guidance on adopting a healthy lifestyle, including the importance of diet, exercise, smoking cessation, and returning to normal activities and work. This information was provided through an interactive mind map accessible via QR codes, aimed at helping patients navigate their recovery process and alleviate anxiety related to their condition.

The comprehensive approach to medication management was part of a broader

strategy to empower patients with the knowledge and tools necessary to take control of their health. By combining education on medication with lifestyle advice, this intervention aimed to improve both adherence to prescribed treatments and overall well-being during recovery.

### *1.3. Nutritional Guidance*

Nutritional support plays a vital role in preventing further cardiovascular events and promoting long-term health. The intervention group received tailored nutritional guidance focused on heart-healthy diets. Nurses provided educational sessions on the importance of a balanced diet, including the reduction of saturated fats, increased intake of fruits and vegetables, and moderation of sodium and sugar. Patients were educated on how dietary modifications could support weight management, blood lipid control, and overall cardiovascular health. In addition, dietary suggestions were personalized according to each patient's health condition and preferences, ensuring feasibility and sustainability.

### *1.4. Psychological Support*

Psychological well-being is a crucial factor in the recovery of cardiovascular patients, especially following a major procedure such as PCI. To address the psychological needs of patients, the intervention group received regular psychological support. Nurses conducted weekly sessions focused on stress management, coping strategies, and emotional well-being. The program included guided relaxation techniques, mindfulness exercises, and educational resources aimed at reducing anxiety, depression, and fear related to recovery. The psychological support sessions were designed to enhance patients’ mental resilience, improve their emotional responses to recovery, and encourage a more positive outlook toward rehabilitation.

### *1.5. Sleep Management*

Poor sleep quality is a common concern for cardiovascular patients, and sleep disturbances can negatively affect recovery outcomes. As part of the intervention, patients received sleep management strategies. Nurses provided education on good sleep hygiene practices, such as establishing a regular sleep schedule, avoiding stimulants before bed, and creating a conducive sleep environment. For patients

reporting persistent sleep difficulties, personalized strategies were developed to help improve sleep quality and promote overall recovery.

### *1.6. Health Education*

Health education was a central component of the rehabilitation program, aimed at empowering patients to actively participate in their recovery. The intervention group received educational materials covering key topics such as disease understanding, PCI recovery, lifestyle modifications, and self-management techniques. Educational content was delivered through videos, images, and written explanations integrated into the digital platform, allowing patients to access information at their convenience. The program used simple, clear language and was accompanied by audio and subtitles in standard Mandarin to ensure accessibility, especially for those with language or literacy barriers.

### *1.7. Smoking Cessation Assistance*

Smoking cessation is essential for improving cardiovascular health and preventing future complications. In the intervention group, nurses provided ongoing smoking cessation support through counseling and educational sessions. Patients were educated on the harmful effects of smoking on heart health and were offered strategies to quit smoking, including behavioral therapy, nicotine replacement options, and motivational support. Progress was monitored regularly, and patients received personalized feedback and encouragement to help them stay on track with their cessation goals.

## *2. Post-Discharge Rehabilitation*

Upon discharge, patients continued to receive individualized follow-up education through WeChat software during the first, second, and third months post-surgery. In addition, patients were scheduled for outpatient follow-up visits during the third months post-surgery. These follow-up sessions allowed the multidisciplinary intervention team to monitor the patients' progress in terms of physical activity, dietary habits, sleep quality, psychological status, and smoking cessation efforts.

Personalized rehabilitation plans were adjusted according to each patient's ongoing needs and progress.

At discharge, patients' families were trained on how to assist in symptom recognition and management, reinforcing the importance of family support in the rehabilitation process. All rehabilitation materials were provided as QR codes to facilitate patient self-learning and easy access to ongoing educational content.

***Development and Quality Control***

The content of the cardiac rehabilitation program package was entirely developed by a multidisciplinary team comprising cardiovascular nurses, clinicians, and rehabilitation physicians. The team systematically reviewed the best available evidence on exercise rehabilitation for coronary artery disease patients and incorporated it into the program. To ensure accessibility and effectiveness, the program utilized simple language, videos, audio, and subtitles in standard Mandarin. Regular quality control procedures and feedback mechanisms were implemented to assess and optimize the program's efficacy throughout the study period.

**Control group**

The control group received only routine nursing care and health education, which was implemented through verbal education combined with the "317 Nursing Education Platform"; routine health education included admission education, preoperative and postoperative education, discharge guidance, and health education at any time during the treatment process. In the control group, no specific individual exercise instructions were provided, especially regarding preoperative and postoperative activities.

**Randomization**

Owing to the nature of the intervention, the participants were not randomly assigned. To minimize potential confounding factors, the multidimensional digital cardiac rehabilitation intervention group and the usual care group were matched on the basis of propensity scores calculated using logistic multiple regression analysis. The matching process involved considering covariates such as age, sex, education level, marital status, exercise habits, monthly salary, current address, living arrangement

(solitary or not), history of CHD, history of PCI, length of hospitalization, medical insurance status and polypharmacy data obtained from presurveys. Propensity score analysis was conducted using a 1-to-1 ratio matching approach with the nearest neighbour method to ensure balanced characteristics between the intervention and control groups. A standard calliper width of 0.2 was used for the propensity score. After matching, a total of 136 patients were included, with 68 patients in the multidimensional digital cardiac rehabilitation group and 68 patients in the usual care group.

**Study Outcome**

All clinical outcome measurements were obtained at the three-month follow-up. The primary outcomes included differences in the 6MWT, the SF-12 scores, and the frailty phenotype scores. Secondary outcomes comprised step speed, 30s-CST, grip strength, WC, BMI and changes in lipid profiles (TC, HDL-C, LDL-C, and TG) at 3 months.

***Physical Fitness***

The assessment of physical fitness parameters, including grip strength, walking speed, and balance, was conducted at two time points: upon admission and at the three-month follow-up. ① Grip strength was measured via an electronic grip device (manufacturer model: Guangdong Zhongshan Xiangshan Scale Instrument Co., Ltd. EH201R). During the measurement, a sitting position was maintained, the upper limbs naturally drooped at the side of the body, the palm faced the body, the grip metre handle faced outwards, the grip posture was adjusted according to palm size of the palm, and the grip metre was forced for measurement. A stable attitude was maintained during the measurement, and the grip metre was not swung back and forth. The grip strength of the dominant hand was measured 3 times, and the maximum value was taken as the final measurement result. ② Step speed: 4.6 m. The investigator used a stopwatch (model: new Dragon XL-011) to measure step speed in "s" (to 2 decimal places). ③ 30-s chair standing test: A straight back chair (sitting height of 43.2 cm) was used, and the patient was informed of the evaluation process and shown the movement before the test. The seat was placed against the wall, and the patient sat in a chair with their back straight, feet flat on the ground, and arms and

wrists crossed and held at the chest. When the test began, the patient completed 1-2 exercises, performed the preexperiment, and then started the timed test. The correct movement was as follows: completing the full standing position and then sitting down. If the patient's buttocks could touch the chair seat position, the above movements were repeated as much as possible within 30 s, and the effective number of movements was recorded. ④ BMI was calculated as follows: BMI = body weight kg/(height m) ^ 2; and waist circumference per cm was measured.

***6-Minute Walking Test***

The 6-Minute Walking Test (6MWT), developed by Bakle, better evaluates the level of exercise tolerance and cardiac rehabilitation by measuring the distance walked in a defined period. The 6MWT was used to test cardiorespiratory function according to the guidelines of the American Thoracic Society. Each participant was asked to walk (not run) back and forth along a corridor as fast as possible to determine the longest possible distance walked within 6 minutes.

***Frailty***

The frailty phenotype (FP) was evaluated according to the following indicators: ① weight loss: weight loss of >5% or >4.54 kg in the past year; ② decreased gait speed (based on the calculation of gait speed according to a 4.57-m walking time): male height ≤173 cm: gait speed ≥7 s; height >173 cm: gait speed ≥6 s; female height ≤159 cm: gait speed ≥7 s; height >159 cm: gait speed ≥6 s; ③ decreased grip strength according to the corresponding sex and BMI range: gait speed ≥7 s; height >159 cm: gait speed ≥6 s; female height ≤159 cm: step speed ≥7 s; height >159 cm: step speed ≥6 s; ③ grip strength: measured according to the lower limit of grip strength within the range of the corresponding sex and BMI and the strength of the dominant hand measured with a grip strength device; ④ self-reported fatigue; and ⑤ physical activity level: male <383 kcal (approximately 2.5 h of walking); female <270 kcal (approximately 2 h of walking). The debilitation was diagnosed if 3 or more indicators were met, and predebilitation was diagnosed if 1 or 2 indicators were met. The FP is mainly used for routine screening of the degree of debilitation in hospitalized patients.

***Quality of Life***

The 12-item Short Form Health Survey (SF-12) was administered in this study. The SF-12 consists of 12 items and includes eight dimensions of general health (GH), physical functioning (PF), vitality status (VT), mental health (MH), somatic pain (BP), somatic role restriction (RP), emotional role restriction (RE), and social functioning (SF). The PF, RP, BP, and VT dimensions compose the physical component summary (PCS), and the GH, SF, RE, and MH dimensions compose the mental component summary (MCS). The scores of each dimension and the total PCS and MCS scores were calculated sequentially, with a total possible score of 100; higher scores indicate better self-perceived quality of life for patients.

Blood lipid indices

The following measurements were taken at hospital admission and during follow-up visits: body weight and height, which were used to calculate lipid values (total cholesterol (TC), high-density lipoprotein cholesterol (HDL-C), low-density lipoprotein cholesterol (LDL-C), and triglyceride (TG) level).

**Statistical Method**

Statistical analysis was performed using R (4.2.0). Continuous variables are presented as the means ± standard deviations if they followed a normal distribution, and differences between groups were assessed via t tests. Alternatively, continuous variables that did not follow a normal distribution are presented as medians (interquartile ranges), and group comparisons were performed using the Wilcoxon test. Categorical variables are reported as frequencies (percentages), and group differences were evaluated using either the chi-square test or Fisher's exact probability method. Statistical significance was considered when $P<0.05$.

## Results

Baseline characteristics were analyzed using propensity score matching. A total of 164 patients were included, with 89 assigned to the multidimensional digital CR intervention group and 75 to the usual care group. After applying a 1:1 matching approach to adjust for baseline inconsistencies, 68 patients were selected from each

group. Statistically, non-significant differences were observed between the two groups after matching baseline data ($P \geq 0.05$). Among 136 patients undergoing PCI (mean age: 70.0 years), 59 (43.38%) were female.

## Discussion

Findings indicated that a multidimensional digital CR intervention improved functional status (FP, 6MWT, gait speed, and 30-s CST) and metabolic markers (TC, TG, and LDL-C) in patients with UA undergoing PCI within a relatively short period compared to standard management. These results emphasize the program's potential to enhance physical recovery and cardiovascular health. Significant time effects were also observed for grip strength, WC, BMI, and overall quality of life (physical and mental components), reinforcing the efficacy of digital interventions. This novel rehabilitation model provides CVD education, clarifies essential diagnostic procedures, provides exercise rehabilitation guidance, and supports home-based rehabilitation. Promoting physical activity, encouraging lifestyle modifications, and facilitating behavioral changes during recovery contribute to improved quality of life in patients with UA undergoing PCI.

## Informed Consent

Multidimensional digital cardiac rehabilitation research trials of recruiting, screening, and into the group of participants in the First Hospital of Bengbu Medical University. We through the electronic system of the hospital and outpatient clinic to determine potential qualified patients, and arrange the next recruitment consultancy. We consulted all potential patients who met the major inclusion criteria on-site at the time of admission. In the process of participants selected, willing to participate in trials of patients will be required to allow to input the information into the database, for the next step screening of patients. Patients is expected at the beginning of the screening visit to conduct a comprehensive screening, to evaluate the qualification test. Patients who pass the screening visit will be considered eligible to enter the informed consent process. During this process, we will provide eligible patients with a detailed verbal description of the study, including its risks, potential benefits, and requirements. We will also provide a paper informed consent form for eligible patients to read, and patients will have ample time to read and reflect on participation. If required, the individual will be given additional time to consider participation, including rearranging the screening visit. Before get written consent, everyone can ask questions, until individual decision making. When ready, participants will be asked to sign the consent form. Then, we will collect the data to provide informed consent of patients, including from the eligible patients' electronic health records and research the information of the program.

# Appendix. Informed Consent

# Informed Consent

**Title:**Effectiveness of Multidimensional Digital Cardiac Rehabilitation in Unstable Angina Patients Undergoing Percutaneous Coronary Intervention: An Emulated Target Trial.

**Informed Consent • Informed Notice page**

**Dear participant,**

We would like to extend an invitation for you to take part in Effectiveness of Multidimensional Digital Cardiac Rehabilitation in Unstable Angina Patients Undergoing Percutaneous Coronary Intervention. This study has undergone a thorough review and has been approved by the Ethics Committee of the First Hospital of Bengbu Medical University.

Before making a decision about participating in this clinical study, we kindly ask you to carefully read the following information. This will help you understand the study's purpose, duration, requirements, and potential benefits, risks, and discomforts associated with participation. Please note that your participation is voluntary, and you have the right to choose whether to participate or not.

You may discuss this study and the information provided with your family, friends, doctor, or other trusted individuals. The study doctor will explain this information to you, and if you have any questions, they will be happy to answer them. If you decide to participate, you will be asked to sign an informed consent form (at the end of this document) before any study-related procedures are performed.

**Introduction**

Unstable Angina (UA), a significant subtype of coronary heart disease (CHD), primarily affects elderly individuals and those with multiple cardiovascular risk factors. It is associated with a high incidence of cardiovascular events, with approximately 20% of cardiovascular disease patients affected by UA. Although advancements in revascularization techniques and preventive measures, such as

intensified lipid-lowering therapies, have improved UA management and reduced acute events, a subset of patients continues to face elevated risks.

Exercise-based cardiac rehabilitation (CR) is a key secondary prevention strategy in cardiovascular disease, playing a vital role in improving muscle strength, endurance, and cardiac function. CR also addresses emotional health, managing anxiety and depression commonly associated with CHD. Despite its proven benefits, participation in CR remains suboptimal, with less than 50% of eligible patients engaging in post-acute coronary event rehabilitation. Contributing factors include short hospital stays, insufficient rehabilitation guidance, and patient-related barriers such as age, comorbidities, and fear of exercise.

Digital health technologies (DHT) offer a promising solution, enabling personalized, low-cost, and scalable cardiac rehabilitation. While DHT has shown potential in enhancing exercise capacity and quality of life, its effectiveness in UA patients undergoing PCI remains underexplored. This study aims to evaluate the impact of digital cardiac rehabilitation on UA patients post-PCI.

## Research Purpose:

**Primary Purpose:** To assess the improvement in 6-minute walk test (6MWT), 12-item Short Form Health Survey (SF-12) sores and frailty phenotype sores at 3 months.

**Secondary Purpose:** To evaluate the changes in gait speed, 30-second chair stand test (30-s CST), grip strength, waist circumference (WC), body mass index (BMI), total cholesterol (TC), high-density lipoprotein cholesterol (HDL-C) levels, low-density lipoprotein cholesterol (LDL-C) levels, and triglycerides(TG) levels at 3 months.

**Inclusion and exclusion criteria.**

| Inclusion criteria |
| --- |
| Age between 18 and 74 years old |
| Patients diagnosed with UA who successfully underwent PCI |
| Patients classified as having cardiac function grades 1 to 3 |
| Patients with no new or recurrent chest pain for at least eight hours after |

| |
|---|
| reperfusion therapy, no new arrhythmias or ECG changes within this timeframe, and no further elevation in myocardial injury marker levels |
| Patients capable of using a smartphone |
| Patients who understood the purpose of this study and voluntarily provided written informed consent |
| Exclusion criteria |
| Previous PCI surgery |
| Out of age |
| Patients with significant postoperative wound bleeding, severe infection, fever, severe electrolyte–acid–base imbalance, severe hypoxia, or malnutrition |
| Patients classified as having cardiac function grades 4 |
| Patients with hepatic and renal failure |
| Patients with severe physical mobility impairments |
| Refusal to sign consent form |
| Mental abnormality |
| Not suitable for participation |

**How many people will participate in this study?**

The plan is to recruit 200 subjects in this study at our institution.

**Study Procedure**

(1) Prior to your inclusion in the study, the research team will collect the following information and examination results to determine your eligibility for participation:

Medical History: The study physician will inquire about any current or past medical conditions.

Demographic Information: The study physician will gather personal information, including your date of birth and ethnic background.

Laboratory Results: Blood biochemistry and complete blood count will be assessed.

Height and Weight: Research personnel will measure your height and weight.

Vital Signs: The study physician will record your blood pressure, heart rate, temperature, and respiratory rate.

Holter Monitor Results: Dynamic electrocardiogram results will be obtained.

Echocardiogram Results: Results from echocardiographic examinations will be collected.

Coronary CT Angiography: Coronary CT Angiography results will be reviewed to assess the extent and severity of coronary artery disease.

Lipid Levels: Blood lipid levels, including total cholesterol, LDL-C, HDL-C, and triglycerides, will be measured to evaluate lipid profile and cardiovascular risk.

(2) If the above information and results meet the eligibility criteria, you will be confirmed for inclusion in the study. You will then be assigned to either the control group or the intervention group, with a treatment duration of 12 week. During this treatment period, researchers will observe the actual effects of the video intervention or control intervention. To ensure accurate recording and assessment of the intervention's effects, your cooperation is required in completing the following assessments:

The 6-minute walk test (6MWT)

• 12-item Short Form Health Survey (SF-12) scores
• Frailty phenotype scores at 3 months
• 30-second chair stand test (30-s CST)
• Grip strength
• Waist circumference (WC)
• Body mass index (BMI)
• Total cholesterol (TC)
• High-density lipoprotein cholesterol (HDL-C) levels
• Low-density lipoprotein cholesterol (LDL-C) levels
• Triglycerides (TG) levels

(3) Upon completing all treatments, researchers will conduct a follow-up via telephone or request that you return for a follow-up visit 12 weeks after treatment completion. During this period, the following assessments will be conducted:

• The 6-minute walk test (6MWT)
• 12-item Short Form Health Survey (SF-12) scores

• Frailty phenotype scores at 3 months
• 30-second chair stand test (30-s CST)
• Grip strength
• Waist circumference (WC)
• Body mass index (BMI)
• Total cholesterol (TC)
• High-density lipoprotein cholesterol (HDL-C) levels
• Low-density lipoprotein cholesterol (LDL-C) levels
• Triglycerides (TG) levels

(4) Upon completion of all follow-ups, you will be considered to have finished the study.

**How long will this study last?**

This clinical trial will last for 3 months, during which time you will receive follow-up phone calls during months 1, 2, and 3 after surgery and an outpatient visit during month 3. You have the right to withdraw from the study at any time without fear of discrimination or retaliation, and your decision to withdraw will not affect your medical treatment or rights. Your clinician or researcher may also suspend your participation in the study at any time if it is deemed to be in your best interest (the reason for possible termination of the trial will be explained to you). If you choose to participate in this study, we kindly request that you commit to completing the entire research process. In the event that you withdraw from the study for any reason, a relevant examination may be conducted to ensure your safety.

**1. Risks and/or discomforts of participating in this study**

There are no risks associated with participating in this study. However, there may be information security risks. We will do our best to protect the information you provide from being disclosed. Some of the questions we ask you in this study may make you feel uncomfortable, and you have the right to refuse to answer such questions. Additionally, you can take a break at any time during the study. At any point during the study, you can choose to withdraw from the study.

**2. Benefits of participating in the study**

If you agree to participate in this study, you may be able to receive direct medical benefits. Specifically, this study is designed to improve your progress toward recovery after PCI. However, we cannot guarantee this outcome. We hope that the information gained from your participation in this study will help to better inform the diagnosis and treatment of UA in the future.

**3. Alternative treatment options if not participating in the study**

This study will not provide any other treatment options. Your diagnosis and treatment will be determined by the research doctor based on your condition, and you can continue with your regular treatment plan.

The use of research results and confidentiality of personal information: In this study, your personal information will be collected for statistical and analytical purposes. You will have the opportunity to learn about the research results. You can ask your research doctor for the results and ask for an explanation. The results of this study may also be published in journals or presented at conferences, but they will not contain any information that could identify you.

To ensure privacy, records or samples published for research purposes will not include your name or any other identifying information. Instead, your information will only be identified by a code. Only the research doctor and authorized personnel can link this code to your name through a list, which will be securely stored at the research center.

In order to ensure that the research is conducted in accordance with regulations, the applicant, ethics review committee, and government regulatory agencies may access your information when necessary. They are bound by confidentiality obligations and will not violate your privacy.

You have the right to control the use and disclosure of your personal information. You can request to view your medical information at any time, as permitted by national law. You have the right.

**Research-related updates**

During the course of the study, if there are any changes to the study protocol or application, your research doctor will immediately inform you and discuss with you

whether you wish to continue participating in the study. If you decide not to continue, your medical treatment and rights will not be affected. If you choose to remain in the study, your research doctor may ask you to sign a new informed consent form.

**Study costs, compensation, and damages**

If you participate in this study, you will not be required to pay any additional fees. You will only be responsible for the costs associated with the relevant medical examinations for your underlying condition. You will not receive any financial compensation for your participation in the study. However, during the follow-up process, you will receive health guidance from our professional medical staff. If you suffer any harm as a result of participating in the study, you will receive professional treatment provided by the department of cardiology of Renmin Hospital of Wuhan University, and will be compensated in accordance with the law.

**Rights and Responsibilities of Participants**

**1. Rights**

Throughout the entire research process, your participation is voluntary. If you decide not to participate in this study, it will not affect any other treatment you may receive. If you choose to participate, you will be asked to sign this informed consent form. You have the right to withdraw from the study at any time without discrimination or unfair treatment, and your medical treatment and rights will not be affected.

**2. Responsibilities**

As a participant in this study, please abide by the following agreements:

• Return to the hospital for scheduled visits on time.

• You can inform your research doctor at any time if you wish to terminate the study.

• Provide truthful information about your medical history and current physical condition.

• Follow the instructions of the research staff.

• Inform the research doctor of any discomfort you experience during the study.

• Any experimental treatment may pose a risk to you or your fetus, so you and

your partner should avoid any activities that may lead to pregnancy during the study. If you become pregnant during the study, please inform your research doctor immediately.

**3. Contact Information**

If you have any questions related to this study, please contact the researcher at 13955217648.

If you have any questions regarding your rights and interests, or if you wish to report any difficulties, dissatisfaction, or concerns during your participation in this study, or if you wish to provide feedback or suggestions related to this study, please contact the Ethics Committee of the First Hospital of Bengbu Medical University at byyfyll@163.com.

**Informed Consent Form • Consent Signature Page**

**Participant Declaration:**

I have been given information about the study's background, purpose, methodology, potential risks, and benefits. I have had ample time and opportunity to ask questions, and I am content with the responses I have received. Additionally, I have been made aware of who to contact if I have any inquiries, concerns, recommendations, or would like to offer additional information or support for the study.

I acknowledge that my participation in this study is entirely voluntary, and I affirm that I have been given ample time to carefully consider and willingly consent to take part. I retain the right to withdraw from the study at any point without fear of any negative consequences or repercussions on my medical treatment or personal rights. Additionally, I have been assured that the researchers have not employed any deceitful tactics, coercion, or undue pressure to compel my participation in the study.

I acknowledge that in the event of my condition deteriorating, or if I encounter severe adverse reactions, or if my research doctor deems that my continued participation in the study is not in my best interest, he/she may withdraw me from the study. Additionally, the sponsor or regulatory agency may terminate the study during

the research period without my consent. In such an event, my doctor will inform me promptly, and my research doctor will discuss alternative options with me.

I have carefully reviewed and understood the contents of this informed consent form, and I willingly consent to participate in this study. I acknowledge that I will be provided with a copy of the original informed consent form, which will include both my and the researcher's signature and the date of signing.

Participant Signature: Date:

Contact Phone Number:

Legal Representative Signature: Date:

Contact Phone Number:

(Note: If the participant has no legal capacity or limited legal capacity, such as inclusion of vulnerable groups with mental disorders/unconsciousness, the legal representative needs to sign at the following legal representative signature)

Fair Witness Signature: Date:

Contact Phone Number:

(Note: Only when it is possible to include participants with decision-making capacity but unable to read the text, such as illiteracy, visual impairment, a fair witness signature is required. The researcher should keep video materials as proof of informed consent when the witness is informed.)

Researcher Declaration:

I have accurately informed the participant of this document, and he/she has read

this informed consent form accurately and confirmed that the participant had the opportunity to ask questions and voluntarily agreed. I have given him/her a signed original of the informed consent form.

Researcher Signature: Date:

Contact Phone Number:
