# Supplementary figures and images for: The Effectiveness of Nurse-Led Multidimensional Digital Cardiac Rehabilitation in Patients With Unstable Angina Undergoing Percutaneous Coronary Intervention: Emulated Target Trial
Source: J Med Internet Res. 2025 Aug 27;27:e75325. doi: 10.2196/75325 (PMC12384693; doi:10.2196/75325)

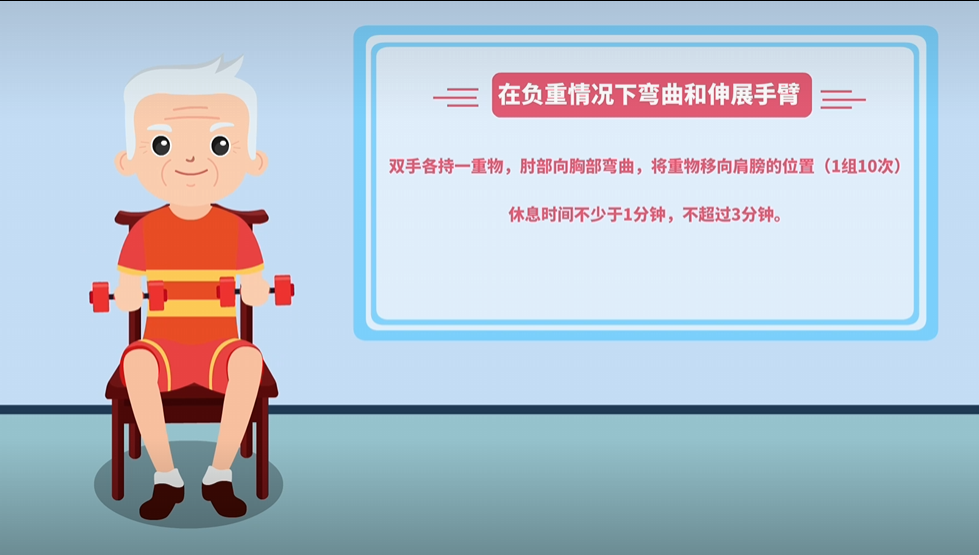

Supplement: Multimedia Appendix 2 [file jmir-v27-e75325-s002.png]
