# Supplementary material for: The Effectiveness of Nurse-Led Multidimensional Digital Cardiac Rehabilitation in Patients With Unstable Angina Undergoing Percutaneous Coronary Intervention: Emulated Target Trial
Source: J Med Internet Res. 2025 Aug 27;27:e75325. doi: 10.2196/75325 (PMC12384693; doi:10.2196/75325)
Supplement: Multimedia Appendix 3 [file jmir-v27-e75325-s003.pdf]

Table S1. Frailty status.

Frailty score	Basline		3-month postdischarge	
	Intervention group	Control group	Intervention group	Control group
0	0 (0%)	0 (0%)	17 (25.00%)	2 (2.94%)
1	41 (60.29%)	43 (63.24%)	45 (66.18%)	46 (67.65%)
2	27 (39.71%)	25 (36.76%)	6 (8.82%)	20 (29.41%)
P	0.86		＜0.001	

Table S2. Comparison of physical fitness in the two groups of patients before and after the intervention.

Variables	Baseline	3-month	F	P	η2G
**Gait speed**					
Control group	0.87（0.14）	0.91（0.15）			
Intervention group	0.87（0.17）	1.05（0.14）			
Group main effect			9.540	0.002	0.066
Time main effect			72.781	<0.001	0.352
Group*time main effect			24.995	<0.001	0.157
**30-s chair stand test**					
Control group	9.76（1.63）	10.00（2.42）			
Intervention group	10.00（1.89）	12.71（1.97）			
Group main effect			25.39	0.001	0.16
Time main effect			66.53	0.001	0.33
Group*time main effect			46.95	0.001	0.26
**Grip strength**					
Control group	17.81（5.80）	17.01（4.51）			
Intervention group	16.64（6.57）	20.74（5.37）			
Group main effect			1.68	0.198	0.01
Time main effect			29.58	0.001	0.18

Group*time main effect			62.65	0.001	0.32
**Waist circumference**					
Control group	93.49（7.69）	93.93（6.40）			
Intervention group	94.98（7.87）	89.91（7.50）			
Group main effect			1.12	0.292	0.01
Time main effect			25.97	0.001	0.18
Group*time main effect			42.10	0.001	0.24
**BMI**					
Control group	24.77（2.70）	24.49（2.48）			
Intervention group	25.74（3.05）	23.88（2.14）			
Group main effect			0.20	0.657	0.00
Time main effect			30.59	0.001	0.19
Group*time main effect			16.82	0.001	0.11
**6MWT**					
Control group	345.15（27.76）	338.15（26.47）			
Intervention group	347.06（32.43）	375.22（29.71）			
Group main effect			16.78	0.001	
Time main effect			46.61	0.001	
Group*time main effect			128.69	0.001	

Note: Data are presented as the mean ± standard deviation.
Abbreviations: BMI = body mass index; 6MWT = 6-minute walking test.

Table S3. Comparative analysis of quality of life (SF-12) in the two groups of patients before and after intervention.

Variables	Baseline	3-month	F	P	η2G
**Physical health**					
Control group	57.22（26.86）	57.45（26.63）			
Intervention group	55.11（23.53）	70.31（18.83）			
Group main effect			1.72	0.192	0.01
Time main effect			143.60	0.001	0.52
Group*time main effect			135.14	0.001	0.50
**Mental health**					
Control group	60.85（21.14）	63.44（18.33）			
Intervention group	58.48（18.59）	71.68（13.78）			
Group main effect			1.04	0.309	0.01
Time main effect			44.75	0.001	0.52
Group*time main effect			20.21	0.001	0.50

Note: Data are presented as the mean ± standard deviation.

Table S4. Comparative analysis of blood lipid in the two groups of patients before and after intervention.

Note: Data are presented as the mean ± standard error.

Variables	Baseline T0	3-month T1	F	P	η2G
**Total Cholesterol**					
Control group	4.51（0.86）	4.51（0.91）			
Intervention group	4.34（0.59）	3.31（0.37）			
Group main effect			36.625	<0.001	0.215
Time main effect			116.687	<0.001	0.465
Group*time main effect			113.188	<0.001	0.458
**Triglyceride**					
Control group	1.71（0.51）	1.68（0.43）			
Intervention group	1.86（0.55）	1.04（0.28）			
Group main effect			20.192	<0.001	0.131
Time main effect			56.813	<0.001	0.298
Group*time main effect			48.218	<0.001	0.265
**Low-density lipoprotein cholesterol**					
Control group	2.01（0.55）	2.24（0.64）			

Intervention group	2.13（0.46）	1.80（0.39）			
Group main effect			7.778	0.006	0.055
Time main effect			0.440	0.508	0.003
Group*time main effect			16.640	<0.001	0.110
**High-density lipoprotein cholesterol**					
Control group	1.13（0.29）	1.14（0.18）			
Intervention group	1.05（0.21）	1.01（0.29）			
Group main effect			9.716	0.002	0.068
Time main effect			0.462	0.498	0.003
Group*time main effect			0.575	0.450	0.004
